# Robot-Assisted Laparoscopic Prostatectomy in a Prostate Cancer Patient Undergoing Continuous Ambulatory Peritoneal Dialysis

**DOI:** 10.1089/cren.2017.0014

**Published:** 2017-04-01

**Authors:** Sohei Kuribayashi, Kentaro Takezawa, Yohei Okuda, Masataka Kawamura, Nozomu Kishimoto, Go Tanigawa, Koichi Tsutahara, Tetsuya Takao, Seiji Yamaguchi

**Affiliations:** Department of Urology, Osaka General Medical Center, Osaka, Japan.

**Keywords:** robot, laparoscopy, prostatectomy, peritoneal dialysis

## Abstract

***Background:*** Robot-assisted laparoscopic prostatectomy (RALP) has become the gold standard treatment for organ-confined prostate cancer. However, no proper surgical approach or appropriate postsurgical management of RALP has been established for a patient undergoing peritoneal dialysis. Here, we present a case of a peritoneal dialysis patient who underwent RALP and reinstated peritoneal dialysis with no trouble associated with peritoneal dialysis.

***Case Presentation:*** The patient was a 61-year-old man with organ-confined prostate cancer. He had been on peritoneal dialysis for 2 years. The peritoneal dialysis catheter was routed subcutaneously from the left lateral region into the abdominal cavity at the paramedian region. RALP was performed by the transperitoneal anterior approach. The surgical maneuver was not influenced by the peritoneal dialysis catheter at all. At the end of surgery, the incised peritoneum was sutured and closed tightly. After surgery, peritoneal dialysis was temporarily interrupted for 2 weeks. Then it was safely reinitiated with no complications.

***Conclusion:*** Transperitoneal RALP with complete peritoneal repair can be a standard treatment option for a prostate cancer patient undergoing peritoneal dialysis.

## Introduction and Background

Prostate cancer, the most common cancer in men, accounts for 25% of all male cancers. Robot-assisted laparoscopic prostatectomy (RALP) has become a gold standard surgery for organ-confined prostate cancer because of its less invasiveness and excellent oncologic and functional outcomes. However, few reports describe RALP for a patient undergoing continuous ambulatory peritoneal dialysis (CAPD). Appropriate approach and perioperative management of RALP are not established for CAPD patients. In general, CAPD patients have a risk of CAPD-related complications such as leakage of dialysate fluid, wound dehiscence, and abdominal hernia. This report describes a case of a CAPD patient who safely underwent RALP by the transperitoneal approach without removal of the CAPD tube. Importantly, we sutured and closed the incised peritoneum tightly, and restarted CAPD effectively 14 days after surgery.

## Presentation of Case

**FIG. 1.**
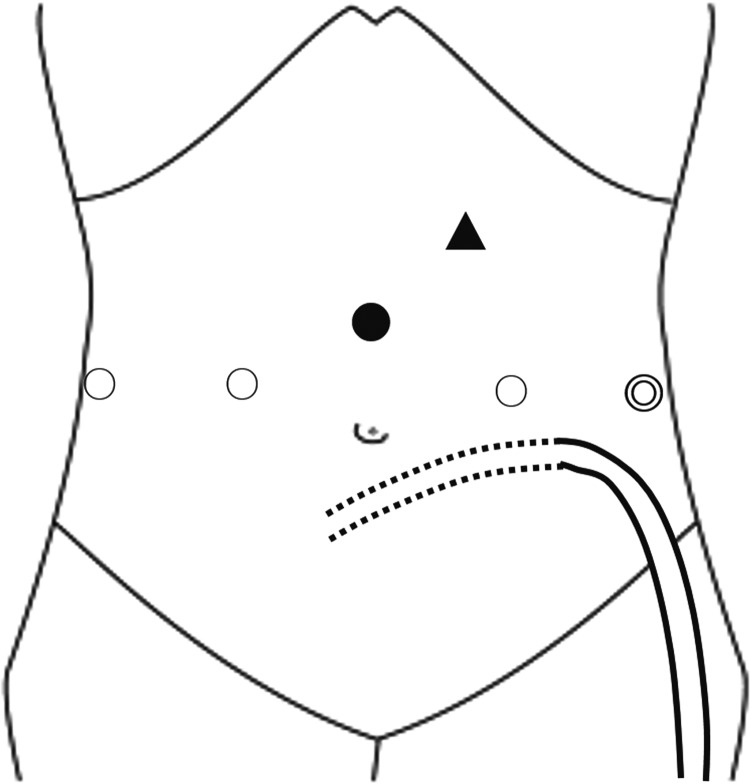
Positions of the trocars and the CAPD tube: 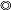 12 mm port for assistant, ○ 8 mm port, ● camera port, and ▲ 5 mm port for assistant. CAPD, continuous ambulatory peritoneal dialysis.

**FIG. 2.**
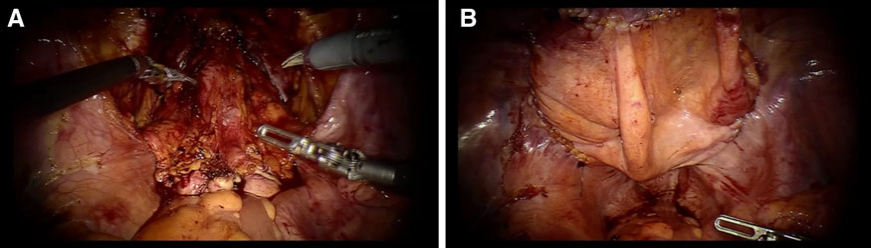
Intraoperative laparoscopic view. **(A)** After the urethrovesical anastomosis and **(B)** after peritoneum repair.

A 61-year-old man who had been on CAPD for 2 years was diagnosed with prostate cancer cT_2a_N_0_M_0_. He underwent RALP. The CAPD tube was routed subcutaneously from the left lateral region into the abdominal cavity at the paramedian region through the infraumbilical portion. RALP was performed with the transperitoneal anterior approach using a four-arm da Vinci Si unit (Intuitive Surgical, Inc., Sunnyvale, CA). Six ports were inserted as usual (Fig. 1). Then the abdominal cavity was insufflated. Although slight adhesion was seen around the left paramedian region and left groin area, it was at a manageable level. The peritoneal end of the CAPD tube was shifted to the upper abdomen area, away from the surgical field. The peritoneum was incised along the umbilical ligaments. The space of Retzius was developed. The prostate gland, seminal vesicle, and vasa were approached and dissected. The operation was entirely unaffected by the CAPD tube. After urethrovesical anastomosis, the drainage tube was placed in the retroperitoneal space. At the end of surgery, the incised peritoneum was sutured and closed tightly with V-loc 180 suture (Covidien, Tyco Healthcare Group, Norwalk, CT) (Fig. 2). The fascias of trocar sites were closed with 2-0 Vicryl (Ethicon, Inc., Somerville, NJ). Pathologic findings were adenocarcinoma, Gleason score 4 + 3, ly0, v0, pn0, sv0, and pT2c. After surgery, hemodialysis was started to interrupt CAPD. The CAPD tube was flushed once a day with heparinized saline until reinstatement of CAPD. The drainage tube was removed 3 days after surgery. Six days after surgery, the urethral catheter was removed after we confirmed from urethrocystography that no leakage had occurred. Fourteen days after surgery, he was started on CAPD with no complications such as dialysate leakage from trocar incision or wound infection. Eight months after surgery, he continued the CAPD with no trouble.

## Discussion and Literature Review

This case demonstrated that RALP can be performed safely for a prostate cancer patient who underwent CAPD. The transperitoneal approach and complete closure of the incised peritoneum are regarded as important in RALP for CAPD patients.

Results show that the transperitoneal approach enables us to perform RALP as usual, even in a CAPD patient. Two approaches exist for RALP: transperitoneal and extraperitoneal. The extraperitoneal approach allows the maintenance of peritoneal integrity, but it requires removal and subsequent reinsertion of the CAPD tube because the CAPD tube makes infraumbilical port placement difficult. The transperitoneal approach does not require the removal of the CAPD tube, but it requires peritoneal incision. Because peritoneal defects engender peritoneal leakage, which may result in absorption of dialysate and inadequate ultrafiltration, the peritoneal incision must be closed completely in CAPD patients. Such repair is rather difficult in conventional laparoscopic surgery. However, robot-assisted surgery greatly simplifies the surgery because of the benefits of three-dimensional imaging and dexterity enhancement. This point represents an important advantage of RALP over conventional laparoscopic radical prostatectomy in CAPD patients. Moreover, we demonstrated that CAPD can be reinstated within 2 weeks after RALP, in which the defect of the peritoneum is repaired completely. No consensus exists about the optimal timing of postoperative reinstatement of CAPD. Reinstatement that is too early will cause CAPD-related complications, including leakage of dialysate fluid, surgical site hernia, and peritonitis.^1^ In the case of open surgery, it has been recommended that CAPD should be restarted 6 weeks after surgery.^1^ Recently, in cases of laparoscopic surgery, early restart of CAPD has been suggested. For cases of laparoscopic cholecystectomy, which is usually performed with 3–4 small incisions, some reports have described that CAPD can be restarted within 2 weeks after surgery. However, peritoneal leakage after laparoscopic surgery has also been reported.^2^ In such cases, the peritoneal injury occurred during retroperitoneoscopic nephrectomy, and peritoneal leakage of dialysis occurred when CAPD was started 2 days after surgery, although the injured peritoneum was sutured tightly.^2^ Based on these earlier reports, we restarted CAPD 2 weeks after operation because we can repair the peritoneum completely. We had no trouble associated with CAPD. Results suggest that we can restart CAPD within 2 weeks after RALP in case the peritoneum is closed tightly. However, additional reports should be accumulated to ascertain whether the timing of reinstatement can be much earlier.

In general, the standard therapy for localized prostate cancer is radical prostatectomy or radiotherapy. Recently, several reports of the relevant literature have described superior overall and cancer-specific survival rates for prostate cancer patients with radical prostatectomy compared with radiotherapy.^3^ It has also been reported that radiotherapy for prostate cancer increases the risk of late secondary malignancies. Moreover, CAPD tends to be selected by younger patients with end-stage renal disease. For example, 67% of CAPD patients are younger than 65 in the United States.^4^ Therefore, RALP may be a preferred treatment option for localized prostate cancer in CAPD patients. However, it is noteworthy that abdominal operation with CAPD patients presents a higher risk of CAPD-related complications such as leakage of dialysate, wound infection, dehiscence, incisional hernia, and catheter failure.^2^ No report of relevant literature describes a study of radical prostatectomy in patients undergoing CAPD. Moreover, the safety and appropriate perioperative management of RALP have remained unclear. In the present case, we showed that RALP can be performed safely with the transperitoneal approach even in a CAPD patient and showed that CAPD can be restarted by complete closure of the peritoneum 14 days after operation. Therefore, RALP can be a standard treatment for organ-confined prostate cancer in CAPD patients.

## Conclusion

Transperitoneal RALP with complete peritoneal repair can be a standard therapy for localized prostate cancer patients undergoing CAPD.

## Abbreviations Used

CAPD: continuous ambulatory peritoneal dialysis
RALP: robot-assisted laparoscopic prostatectomy
